# Advanced Case of Cardiac Amyloidosis Presents With Chronic Diarrhea

**DOI:** 10.7759/cureus.26757

**Published:** 2022-07-11

**Authors:** Mahmoud Abdelsamia, Osama Mosalem, Yasser Radwan, Manal Boumegouas, Heather Laird Fick

**Affiliations:** 1 Internal Medicine, Michigan State University, East Lansing, USA; 2 Cardiovascular Disease, Michigan State University, East Lansing, USA; 3 Internal Medicine, Edward W (EW) Sparrow Hospital, Lansing, USA; 4 Medicine, Michigan State University College of Human Medicine, East Lansing, USA

**Keywords:** amyloidosis al, diastolic heart failure, chronic diarrhea, gi amyloidosis, infiltrative cardiomyopathy

## Abstract

Late diagnosis of light chain (AL) amyloidosis can lead to catastrophic consequences on the quality of life of affected patients and overall disease prognosis. Therefore, clinicians should have high suspicion and recognize clinical red flags for amyloidosis. This case report presents a 65-year-old female who presented to the emergency department with chronic diarrhea and significant weight loss with significant hypotension. The patient was treated four weeks prior to admission with a five-day course of nitrofurantoin for urinary tract infection. The initial workup was positive for Clostridium difficile(C.diff), which was treated medically; however, the patient started to complain of mild shortness of breath accompanied by mildly elevated brain natriuretic peptide (BNP). Later on, the patient had a cardiac arrest and was appropriately resuscitated. Subsequent ECHO showed significant left ventricular hypertrophy, raising high suspicion of myocardial infiltration. Because of persistent diarrhea despite aggressive medical management and an inconclusive workup, the patient underwent colonoscopy with duodenum biopsy, which revealed amyloid deposition confirmed by Congo red staining. The patient afterward suffered from a stroke and recurrent syncopal episodes requiring critical care admission. Due to a compromised quality of life, the patient eventually opted for hospice care. In view of insufficient prospective data spotlighting AL amyloidosis, all patients should be treated within clinical trials whenever possible and ideally evaluated for autologous hematopoietic cell transplantation (HCT) eligibility.

## Introduction

Amyloidosis is a general term that encompasses many subtypes. It is a disorder caused by the extracellular deposition of highly organized fibrils, which can lead to organ dysfunction depending on the type, location, and amount of amyloid disposition. Amyloidosis can range from localized amyloidosis to systemic amyloidosis. We so far know that 18 proteins result in systemic amyloidosis and 22 as localized forms [1]. Some types tend to affect a single predominant organ (i.e., wild-type transthyretin (ATTR) amyloidosis and the heart) and others might affect multiple organs on presentation (i.e., light-chain (AL) amyloidosis) [2]. Chronic diarrhea is a common presenting symptom in adults with various underlying etiologies. Gastrointestinal (GI) amyloidosis should be highly considered in old patients with chronic diarrhea and weight loss, especially when an extensive workup can’t determine the etiology of diarrhea.

## Case presentation

A 65-year-old female with no significant medical history presented to the emergency department (ED) with diarrhea, weakness, and weight loss. She reported having watery diarrhea for at least one year, twice or thrice daily, with no hematochezia, abdominal pain, or vomiting. This was associated with a weight loss of 60 pounds (lbs) over the last year. She said that she couldn’t see a provider except for tele-visits due to the coronavirus disease 2019 (COVID-19) pandemic. Four weeks prior to admission, she was prescribed nitrofurantoin for urinary tract infection. However, she endorsed worsening diarrhea after the antibiotic course associated with dizziness and significant weakness.

In the ED, her blood pressure was 84/55 mmHg; otherwise, she was vitally stable. Labs were significant for hemoglobin (Hb) 8.4 g/dL, mean corpuscular volume (MCV) 86 fL, sodium (Na) 129 meq/L, blood urea nitrogen (BUN) 33 mg/dL, and creatinine 1.37 mg/dL, Other pertinent labs showed ferritin 294 ng/mL, iron 23 ug/dL, sat 7%, and normal total iron-binding capacity (TIBC). Thyroid-stimulating hormone (TSH) was normal. TTG was normal; however, her serum IgA was low. Further workup showed Clostridium difficile (C.diff) colitis, and she was started on oral vancomycin along with aggressive fluid hydration.

**Figure 1:**
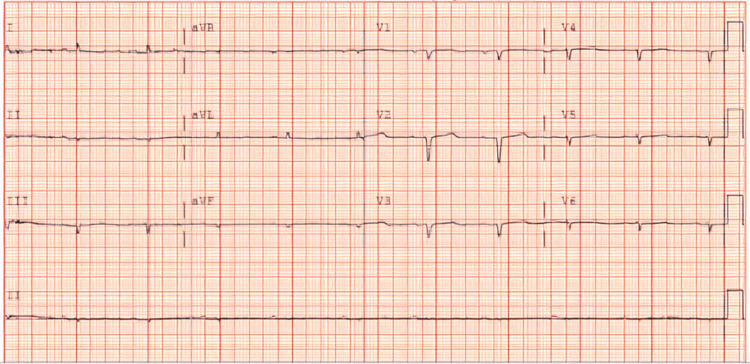
Twelve-lead electrocardiogram showing a heart rate of 60 beats/min, sinus rhythm, and low voltage QRS in the limb leads

**Figure 2:**
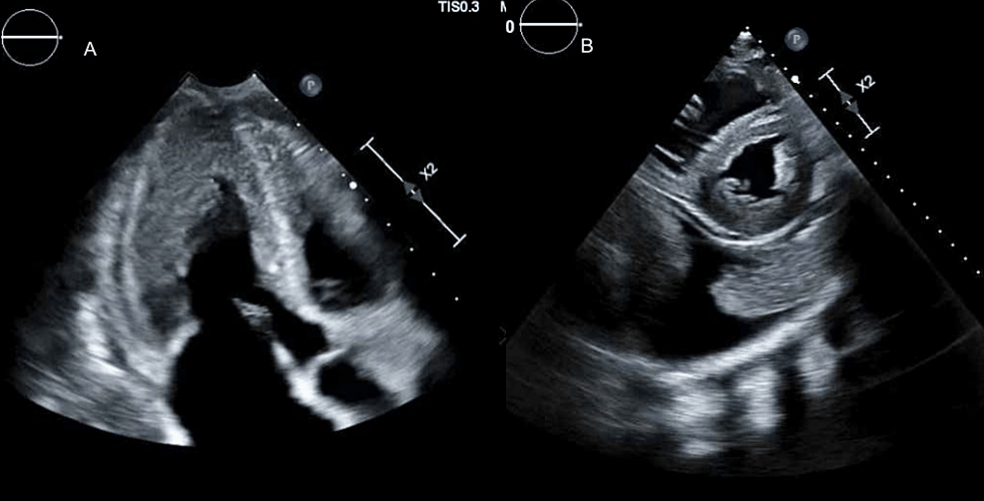
Echocardiography in cardiac amyloidosis A. Showing right ventricular hypertrophy. B. Left ventricular hypertrophy with small pericardial effusion

During her hospitalization, the patient had a profound vasovagal syncope. Shortly after, she had a bowel movement and was noticed to be bradycardic and hypotensive and later on went into cardiac arrest. Cardiac resuscitation was done per advanced cardiac life support protocol, and she regained consciousness after two rounds of cardiopulmonary resuscitation (CPR). An electrocardiogram (ECG) showed sinus rhythm with a heart rate of 60 beats/minute and extremely low voltage ECG (Figure 1). An echocardiogram (ECHO) revealed a left ventricular ejection fraction (LVEF) of 55-60%, left mid and apical ventricular hypertrophy, right ventricular dilation with hypertrophy, and small pericardial effusion concerning infiltrative disease (Figure 2). Gastrointestinal service was consulted for persistent diarrhea out of proportion to C.diff treatment, and they recommended both colonoscopy and endoscopy to be done as an outpatient. Meanwhile, the patient was treated with cholestyramine for her symptomatic diarrhea. An outpatient cardiac pyrophosphate (PYP) scan was arranged, and the patient was discharged to a subacute rehabilitation facility. Immunoglobulin workup that was pending at the time of discharge revealed Kappa free light chain (FLC) 1 mg/dL, lambda FLC 14 mg/dL, and kappa/lambda FLC ratio 0.0714, which is in line with serum protein electrophoresis (SPEP) that showed IgG lambda monoclonal gammopathy.

**Figure 3:**
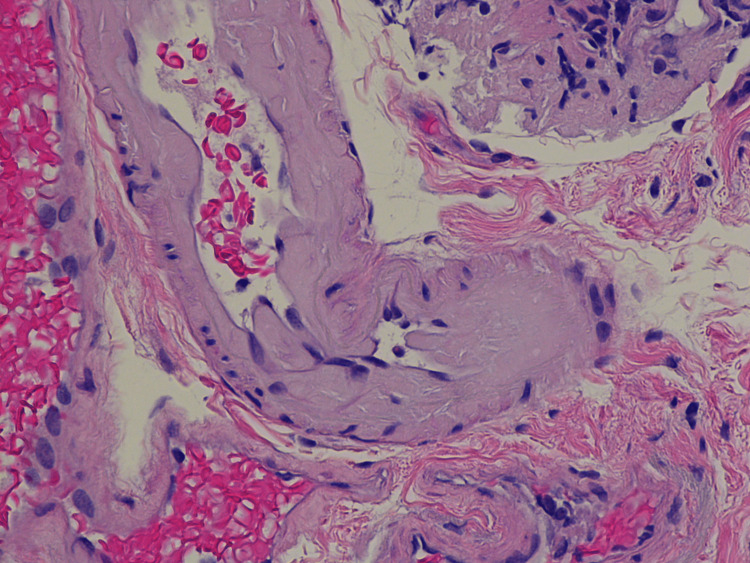
Duodenal mucosal biopsy finding with Congo red staining positive for amyloid Original magnification x200

**Figure 4:**
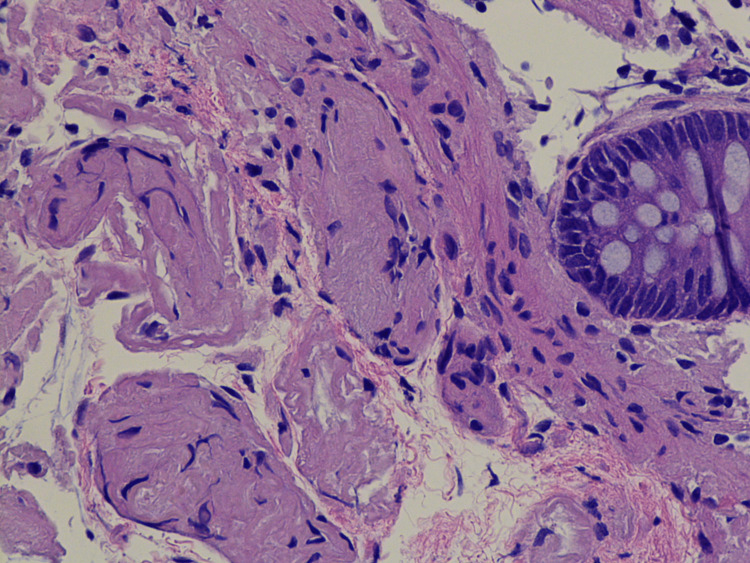
Colon mucosal biopsy with Congo red staining positive for amyloid Original magnification x200

**Figure 5:**
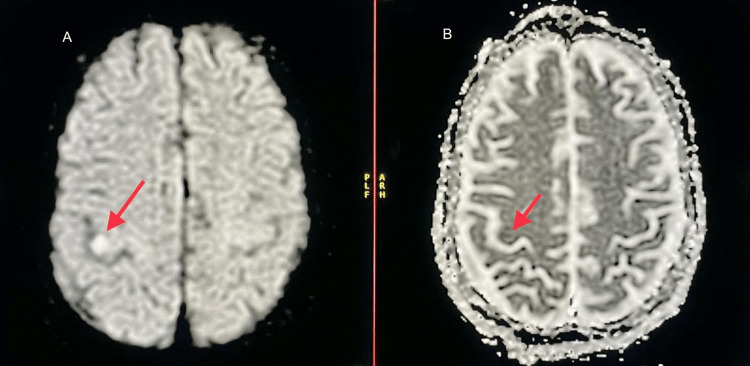
A. Diffusion-weighted imaging demonstrating hyperintensity in the precentral gyrus in the right frontal lobe. B. Apparent diffusion coefficient imaging demonstrating visible but decreased values of hypointensity in the same region

Two weeks later, the patient presented to the hospital with altered mental status and was found to have a urinary tract infection that was appropriately treated with levofloxacin per culture sensitivity. Due to new findings of monoclonal gammopathy highly concerning for amyloidosis and persistence of her diarrhea, the patient underwent colonoscopy and upper endoscopy with grossly unremarkable findings. Pathology revealed Congo red positivity in both duodenal (Figure 3) and colon (Figure 4) biopsies, consistent with the diagnosis of amyloidosis. Despite treatment of her UTI, patient mentation did not improve, which necessitated the involvement of stroke service. MRI brain was obtained that showed focal acute/subacute infarct involving a portion of the precentral gyrus of the right frontal lobe (Figure 5). Computed tomography (CT) of the head and neck was negative. The patient was started on aspirin and statin, and a loop recorder was implanted, as there was no evidence of atrial fibrillation on telemetry. The patient continued to slowly improve and eventually was discharged to a subacute rehabilitation facility.

Outcome/follow-up

The patient did follow up with the oncology service and different diagnostic modalities and potential treatment options were discussed with the patient. It was decided to proceed with a bone marrow biopsy. Unfortunately, the patient was admitted the same day to the intensive care unit with hypotension, bradycardia, and hypothermia. During her stay in the ICU, the patient required vasopressors and later on was weaned to high-dose midodrine in an attempt to maintain adequate blood pressure. She, however, continued to be hypotensive with worsening in her kidney function that was thought to be secondary to hypotension versus possible renal amyloidosis. Goals of care discussion started per patient and family request. Ultimately, she opted for hospice-only measures and the patient was discharged home with home hospice care.

## Discussion

The incidence of immunoglobulin light chain (AL) amyloidosis is 12 cases per million persons per year [3]. It is estimated that the average age at diagnosis is 63 years, and almost 90% of patients are over 50 years [4]. It is also noted that around 69% of patients will have more than one organ involved at diagnosis time [5]. Although several organs might be involved, survival is mainly determined by the extent of heart involvement [6]. Congestive heart failure remains the most recognized cause of death in primary amyloidosis [7]. It is associated with a median survival of fewer than six months and five-year survival of less than 10% [8]. GI amyloidosis is usually rare in primary amyloidosis, with a prevalence of about 1% in a retrospective study [9]. GI amyloidosis can present with a wide array of symptoms. The most observed symptoms were gastrointestinal bleeding, weight loss, malabsorption, and diarrhea [10].

Diagnosis of amyloidosis can be challenging and could be missed resulting in late diagnosis and subsequently delaying treatment initiation. This is highlighted in our case as delayed diagnosis led to detrimental effects on the patient’s quality of life with multiple organ involvement. In a follow-up survey that was completed by 533 participants, the diagnosis of amyloidosis was not established until ≥ 1 year, and it sometimes took more than five physicians to establish a diagnosis [11]. That makes it essential and crucial for timely diagnosis of amyloidosis, which requires the integration of history clues and pertinent physical exam findings in addition to a high degree of suspicion since amyloidosis can present with a wide array of non-specific symptoms. When amyloidosis is suspected, it is practical to start with detecting monoclonal protein either with serum or urine immunofixation besides the FLC level with an almost 99% chance of identifying monoclonal abnormality [12] that would reflect underlying plasma cell dyscrasia, subsequently identifying the amyloid structure by a histological evaluation of the affected organ or accessible surrogate location as abdominal fat aspirate or bone marrow biopsy, Once the amyloid diagnosis is established, it is imperative to identify protein subunit involved [13].

Treatment is aimed directly toward suppressing amyloid production, decreasing the concentration of toxic light chains, and hence, improving organ dysfunction. Early diagnosis and therapy initiation has been shown to decrease mortality and improve survival [14]. Due to the limitation in prospective randomized trials that solely focus on novel treatments for AL amyloidosis, patients with AL amyloidosis should be treated within clinical trials [15]. All patients with newly diagnosed AL amyloidosis should be assessed for autologous hematopoietic cell transplantation (HCT) eligibility. The available literature has shown the superiority of HCT in comparison to conventional chemotherapy treatment alone [16]. It is, however, estimated that over 80% of patients at diagnosis will not be eligible for HCT for various reasons [17]. Induction therapy with Bortezomib-based regimens is widely adapted, and the addition of daratumumab has shown higher frequencies of hematological complete response and survival benefit based on the result of the ANDROMEDA clinical trial [18]. For patients with relapsed or refractory disease, ongoing trials and studies include different treatment approaches to determine the most beneficial therapy for this group of patients. Treatment options include treatment with proteasome inhibitor-based regimens (Ixazomib) and immunomodulatory derivative-based regimens [19-20].

## Conclusions

AL amyloidosis is a rare disease and typically involves multiple organs at presentation. Although GI amyloidosis is a rare cause of chronic diarrhea, it should be considered in elderly patients with chronic unexplained diarrhea, especially when accompanied by other common features of amyloidosis such as hypotension and low-voltage ECG tracing. Cardiac involvement is the leading cause of death in most patients. The available literature has shown that delayed diagnosis leads to a detrimental outcome in patients' prognosis. After the amyloid diagnosis, patients should be assessed for HCT eligibility and treated within clinical trial whenever feasible. Bortezomib-based regimens and the addition of daratumumab have shown promising results in achieving deep hematological remission.
